# Very low-depth sequencing in a founder population identifies a cardioprotective *APOC3* signal missed by genome-wide imputation

**DOI:** 10.1093/hmg/ddw088

**Published:** 2016-05-04

**Authors:** Arthur Gilly, Graham Rs Ritchie, Lorraine Southam, Aliki-Eleni Farmaki, Emmanouil Tsafantakis, George Dedoussis, Eleftheria Zeggini

**Affiliations:** ^1^Wellcome Trust Sanger Institute, Wellcome Genome Campus, Hinxton, Cambridgeshire CB10 1SA, UK; ^2^European Molecular Biology Laboratory, European Bioinformatics Institute, Wellcome Trust Genome Campus, Hinxton, Cambridge CB10 1SD, UK; ^3^Wellcome Trust Centre for Human Genetics, Oxford OX3 7BN, UK; ^4^Department of Nutrition and Dietetics, School of Health Science and Education, Harokopio University, Athens 17671, Greece; ^5^Anogia Medical Centre, Anogia 740 51, Greece

## Abstract

Cohort-wide very low-depth whole-genome sequencing (WGS) can comprehensively capture low-frequency sequence variation for the cost of a dense genome-wide genotyping array. Here, we analyse 1x sequence data across the *APOC3* gene in a founder population from the island of Crete in Greece (*n* = 1239) and find significant evidence for association with blood triglyceride levels with the previously reported R19X cardioprotective null mutation (β = −1.09,σ = 0.163, *P* = 8.2 × 10^−11^) and a second loss of function mutation, rs138326449 (β = −1.17,σ = 0.188, *P* = 1.14 × 10^−9^). The signal cannot be recapitulated by imputing genome-wide genotype data on a large reference panel of 5122 individuals including 249 with 4x WGS data from the same population. Gene-level meta-analysis with other studies reporting burden signals at *APOC3* provides robust evidence for a replicable cardioprotective rare variant aggregation (*P* = 3.2 × 10^−31^, *n* = 13 480).

## Introduction

Dyslipidaemia is a well-established risk factor for cardiovascular disease, the leading cause of death worldwide. Blood lipid levels have a heritable component, and the underlying common-frequency genetic determinants have been studied in large-scale genome-wide association studies (GWAS) (1,2). Apolipoprotein CIII plays an important role in regulating triglyceride (TG) levels (3). Common-frequency variants upstream of the *APOC3* gene, coding for apolipoprotein CIII, have been associated with plasma TG levels at genome-wide significance in studies of ∼100 000 individuals (2). More recently, a rare splice variant in *APOC3* was found to be associated with blood TG levels in the UK10K study, replicating across a total of ∼15 000 European individuals (4). Power to detect genetic associations can be considerably higher in isolated populations as rare variants may have drifted up in frequency following the bottleneck event (5,6). In 2008, a low-frequency *APOC3* null mutation (R19X) was found to have a cardioprotective effect in the Amish founder population (*n* ∼ 1800) (7), and the same variant was subsequently found to be associated with reduced TG levels in an isolated Greek population (*n* ∼ 1000) (8). R19X has independently risen in frequency to over 1% in both isolates, and is very rare (∼0.05%) in the general European population.

A burden of rare loss of function (LoF) variants in *APOC3* was found to be associated with coronary heart disease and TG levels in the Exome Sequencing Project study across ∼110 000 individuals from cosmopolitan populations (9). Recently, exome sequencing of ∼8500 European American and African American individuals identified a rare LoF variant burden in *APOC3*, also associated with TGs (10). Here, we use very low-depth whole-genome sequencing (WGS) data in a Greek isolated population to describe an *APOC3* cardioprotective signal missed by genome-wide imputation and to provide empirical proof-of-principle of how very low-depth sequencing can leverage the power advantages afforded by founder populations in catalysing these discoveries.

## Results

**Figure 1.**
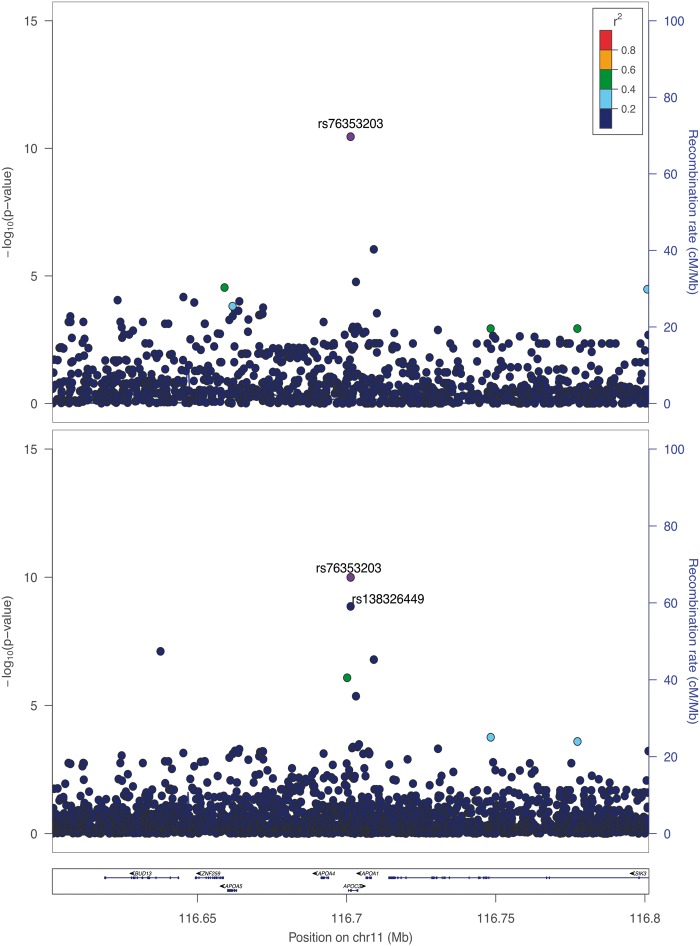
Regional association plots for TGs in the *APOC3* region (*n* = 1225 overlapping samples). Top panel: MANOLIS GWAS data imputed up to a merged reference panel including MANOLIS, UK10K and 1000 Genomes Project WGS. Bottom panel: MANOLIS low-depth WGS data. LD values were derived using genotype data from each dataset.

A total of 990 individuals from the Hellenic 20 Isolated Cohorts - Minoan Isolates (HELIC-MANOLIS) study were sequenced at 1x depth and 249 at 4x depth using Illumina HiSeq (total 1239 samples). Following variant calling and imputation-based genotype refinement, we identified 57 single nucleotide variants (SNVs) in the *APOC3* gene (Supplementary Material, Table S1). We performed single-point association analysis with TG levels (*n* = 1192), using a threshold of 1 × 10^−^^8^ to define genome-wide significance. Two variants exceeded this threshold, the null mutation R19X (rs76353203, β = −1.09,σ = 0.163, *P* = 8.2 × 10^−^^11^), which is a C/T substitution in exon 2 that changes codon 19 into a premature stop codon, and the splice donor variant rs138326449 (β = −1.17,σ = 0.188, *P* = 1.14 × 10^−^^9^), located 1 base pair downstream, which disrupts the donor splice site in intron 2. These two variants are in very low linkage disequilibrium (LD) (*r*^2 ^< 0.0001) (Fig. 1).

To confirm genotype calling and imputation accuracy, we genotyped both R19X and rs138326449 in a subset of 1087 individuals using Sequenom massARRAY technology. In total, 98.9% of all genotypes were concordant for R19X and 99.1% for rs138326449. Minor allele concordance reached 72.2 and 80%, respectively. The fraction of true positives among non-reference calls, or positive predictive value (PPV) was high for both variants (96.3 and 100%), indicating that most mismatches were caused by false negatives rather than overconfidence in calling the alternate allele. We repeated the association analysis using the directly genotyped samples (*n* = 1087), and found both variants to remain significantly associated with TG levels (β = −1.19,σ = 0.165, *P* = 3.24 × 10^−^^12^ for R19X; β = −1.10,σ = 0.190, *P* = 1.63 × 10^−^^8^ for rs138326449), further confirming the validity of this signal.

For burden testing, we restricted our focus on the four potentially functional rare or low-frequency [minor allele frequency (MAF) < 5%] variants that reside in exons or the essential splice sites in the consensus splice variant of *APOC3* (*APOC3-001*) (Table 1). These included the two LoF variants R19X and rs138326449. We additionally identified a single carrier of a novel missense variant (11:116701489) also in codon 19 but in exon 3 as the intron falls between the first and second bases of the codon (Supplementary Material, Fig. S1). The resulting amino acid substitution (R19L) is predicted to be deleterious by SIFT (11) and is not observed in 1000 Genomes Project phase 3 (12), Exome Sequencing Project (13) or Exome Aggregation Consortium data (exac.broadinstitute.org, last accessed April 29, 2016. Exome Aggregation Consortium (ExAC), Cambridge, MA. *accessed April 2015*). Lastly, rs187628630 was also included in the burden, as it resides in the 3’ untranslated region (UTR) of *APOC3-001*. The burden test was performed using SKAT (14) on the set of four variants, and yielded significant evidence for association with decreased TG levels (*P* = 3.0 × 10^−^^18^) (Table 1). Evidence for a burden of rare variants remained strong after removal of R19X from the model (*P* = 6.15 × 10^−^^10^). R19X is not in LD with rs138326449, 11:116701489 or rs187628630, therefore this burden constitutes an independent association not driven by R19X. When rs187628630 and 11:116701489 are removed from the model, the significance of the association with TGs stays unchanged (*P* = 4.3 × 10^−^^18^), but when both R19X and rs138326449 are removed, the association is fully attenuated (*P* = 0.49).

**Table 1.** Association between rare variants in *APOC3* and blood lipid levels

rsID	position	HGVS	N. carriers	Mean TG level	Mean HDL level	MANOLIS MAF (%)	1KG P3 EUR MAF (%)	ESP-EA MAF (%)	ExAC MAF (%)	Single point *P*-value
rs76353203	116701353	p.Arg19Ter, p.Arg37Ter	34	0.847	1.684	1.42	—	0.03	0.07	8.2 × 10^−11^
rs138326449	116701354	c.55 + 1G>A, c.109 + 1G>A, n.87 + 1G>A	28	0.877	1.565	1.17	0.30	0.18	0.14	1.14 × 10^−9^
—	116701489	c.56G>T, p.Arg19Leu	1	1.424	0.932	0.04	—	—	—	0.942
rs187628630	116703739	c.*139C>G	5	1.226	1.497	0.16	0.40	—	—	0.408
Total *APOC3* carriers			67	0.901 (±0.396)	1.598 (±0.363)					
Total *APOC3* non-carriers			1125	1.657 (±1.206)	1.261 (±0.345)					
Carriers v non-carriers (%)				−45.6	+26.7					
*P*-value				3.0 × 10^−18^	4.8 × 10^−16^					

rsID, NCBI dbSNP identifier for the variant; position, position on chromosome 11 in the GRCh37 assembly; HGVS, Human Genome Variant Society nomenclature for each variant; N. carriers, number of carriers of effect allele in MANOLIS. Mean TG level, mean TG levels in carriers, expressed in mmol.l^−1^. Mean HDL level, mean high-density lipoprotein levels in carriers, expressed in mmol.l^−1^. Numbers in parentheses denote standard deviations. *P*-values are calculated using SKAT on sex-stratified log-transformed values for TG, and on sex-stratified, inverse-normal transformed, age and age-squared adjusted values for HDL. MANOLIS MAF, minor allele frequency (MAF) in MANOLIS; 1KG P3 EUR MAF, MAF in the EUR (European) population from phase 3 of the 1000 Genomes Project; ESP-EA MAF, MAF in the EA (European-American) population from the Exome Sequencing Project; ExAC MAF, MAF in all samples from the Exome Aggregation Consortium (all external resources were accessed in April 2015). Single-point *P*-value is the score test *P*-value calculated using GEMMA on sex stratified and log-transformed TG levels.

Both loss-of-function variants, but none of the other variants identified in HELIC-MANOLIS, are included in the rare variant burden associations with TG levels discovered in *APOC3* by two recent large-scale exome sequencing studies. The first, by Crosby *et al.* (9) from the TG and high density lipoprotein (HDL) Working Group of the Exome Sequencing Project, includes two other variants, a missense variant, A43T, in exon 3 (position 116701560), and another splice variant at the donor splice site of intron 3 (position 116701613). The second, by Li *et al.* (10), includes rs140621530, a rare splice donor variant, and the novel singleton frameshift indel 11:116703578. These four variants are all absent from the HELIC-MANOLIS cohort. These differences demonstrate the expected allelic heterogeneity underlying rare variant burden signals that traverse populations and highlight the importance of seeking replication at the locus rather than at the constituent variant level. A gene-level meta-analysis of *APOC3* burden signals across the exome sequencing study by Crosby *et al.* (9), the MANOLIS WGS finding described here and the exome sequencing study by Li *et al.* (10) using Stouffer’s method yields strong evidence for association with TG levels (*P* = 3.23 × 10^−^^31^, *n* = 13 480). Inhibition of apolipoprotein C-III in pre-clinical and clinical studies has been shown to reduce plasma TGs, a major risk factor for cardiovascular disease (15), thereby opening possibilities for new therapeutic routes.

In this work, we used very low-depth WGS to access a greater proportion of the allele frequency spectrum compared with dense GWAS chips at a fraction of the cost of deep WGS. With the advent of ever-increasing imputation reference panel sizes, we explored whether the *APOC3* signal we identified using sequence data could be recapitulated using a hybrid GWAS and imputation approach. Illumina OmniExpress and ExomeChip platform data for 1265 samples, of which 1225 (1178 with a TG level measurement) overlapped with the sequenced samples, were merged and imputed up to an in-house reference panel constructed with IMPUTE2 (13,16). The reference panel contained the phased haplotypes of 1092 samples from the 1000 Genomes Project Phase 1 study,^12^ 3781 7x WGS samples from the UK10K (17) TwinsUK (18) and ALSPAC (19) studies, and 249 MANOLIS samples whole genome sequenced at 4x depth.

Despite being imputed up to a large reference panel including WGS from the same founder population, the only signal above genome-wide significance in the imputed GWAS dataset is R19X (*P* = 3.48 × 10^−^^11^), which is directly typed on the ExomeChip array, but absent from the combined reference panel. Three of the four rare variants included in the sequence-based burden test are present in the imputed data, with the exception of 11:116701489. Imputation quality scores for rs138326449 and rs187628630 are 0.49 and 0.70, respectively, and their association *P*-values for TGs are 0.045 and 0.19 (2.23 × 10^−^^3^ and 0.24 for HDL). The lipid-associated burden of these three variants (*P* = 6 × 10^−^^13^) is fully attenuated when R19X is removed (*P* = 0.11, nine orders of magnitude higher compared with the low-depth sequence data).

## Discussion

As we enter the era of WGS, several challenges associated with design strategies for well-powered cost-effective studies are starting to emerge. In this study, we exemplify the potential power gains conferred by studying founder populations in sequence-based studies, in this case achieving robustly replicating genome-wide significant evidence for association between a medically relevant trait and multiple rare variants with a sample size of ∼1200. We demonstrate that very low-depth sequencing empowers the detection of rare variant signals that can be missed by hybrid genotyping and imputation approaches, even if the imputation panel includes population-specific haplotypes. Going forward, higher-depth WGS will provide a more comprehensive picture of rare variation and enable researchers to explore the as yet untapped landscape of rare variant associations.

## Materials and Methods

### Cohort details

Blood samples were taken for DNA extraction and laboratory-measured lipid levels measurement on 1244 individuals from the Mylopotamos mountainous villages (HELIC-MANOLIS) on the island of Crete. Blood lipids were assessed using enzymatic colorimetric assays including total cholesterol (cholesterol oxidase—phenol aminophenazone method), HDL-cholesterol and TGs (glycerol-3-phosphate oxidase—phenol aminophenazone). The study was approved by the Harokopio University Bioethics Committee, and informed consent was obtained from all subjects. The Mylopotamos villages, which include Anogia, Zoniana, Livadia and Gonies (estimated population size of 6000 in total), have remained geographically isolated for an estimated 1000 years. The genetic isolatedness and demographic history of this population has been established previously (20).

### Sequencing

Nine hundred and ninety-five samples were sequenced at 1x depth and 249 samples at 4x depth using Illumina HiSeq 2000 and Illumina HiSeq 2500 sequencers. Both datasets were then converted to the BAM format and aligned separately using BWA (21). The 4x dataset was mapped to the 1000 Genomes phase 1 reference assembly (g1k) and the 1x dataset to the 1000 Genomes phase 2 reference assembly with decoy sequences (hs37d5). Optical and PCR duplicates were removed using Picard MarkDuplicates and both mapped datasets were merged.

### Variant calling and imputation

Following variant calling using samtools mpileup (22), variant quality score recalibration was performed using Genome Analysis Toolkit - Variant Quality Score Recalibration v.2.7.2 (23–25). We filtered variants to an estimated type-I error of 10% and an estimated type-II error of 1%. Five ethnic outliers were then excluded using PCA analysis performed in R. We ran imputation-based genotype refinement on the entire dataset (*N* = 1239) using Beagle v.4 (26) and a merged 1000 Genomes phase 1 v.3, UK10K and HELIC-MANOLIS 4x WGS panel. Finally, Beagle v.4 was used again to perform imputation and phasing of the non-overlapping positions.

### Single-point association results

After phenotype QC, 1192 TG values remained in the sample. TG levels in mmol.l^−^^1^ were sex-stratified, log-transformed and converted to standardized *z*-scores. Single-point analysis was then performed using GEMMA (27) v.0.94 using a relatedness matrix calculated with the same software on LD-pruned, MAF-filtered (1%) genome-wide variants that satisfied the Hardy-Weinberg exact test (*P* = 1 × 10^−^^5^). Analysis was restricted to the *APOC3* gene region given by Ensembl, and examined suggestive association signals as well as existing loci associated with lipid traits.

We noted the presence of the common intronic variant rs5130 (MAF 15.2%), which is not mentioned in the literature and is absent from the Global Lipids Genetics Consortium (28) dataset but displayed suggestive association in MANOLIS (*P* = 4.37 × 10^−^^6^). A common synonymous variant in exon 3, rs4520, has previously been implicated in hyperlipidaemia in a small-scale study (28), but is not associated with TG levels (*P* = 0.55) and is in low LD with all predicted loss-of-function (LoF) variants in *APOC3* (*r*^2 ^= 0.005 with R19X, *r*^2 ^= 0.0009 with rs138326449 and *r*^2 ^= 0.0009 with 11:116701489) in MANOLIS. We identified one further variant in the 3’ UTR in exon 4, rs5128, which has previously been associated with lipid levels in a candidate gene study in the Hutterite founder population (29). rs5128, a common variant (MANOLIS MAF 10.9%), displays suggestive association with TGs (*P* = 4.17 × 10^−^^4^) but is in very low LD with functional variants (*r*^2 ^= 0.005 with R19X, *r*^2 ^= 0.001 with rs138326449, *r*^2 ^= 0.0002 with 11:116701489). These low LD figures suggest that these early common-frequency associations reported in the literature were not driven by linkage between the reported variants and one or other loss-of-function variants within *APOC3*.

## Supplementary Material

Supplementary Material is available at *HMG* online.

Supplementary Data
