# Supplementary material for: Very low-depth sequencing in a founder population identifies a cardioprotective APOC3 signal missed by genome-wide imputation
Source: Hum Mol Genet. 2016 May 4;25(11):2360–5. doi: 10.1093/hmg/ddw088 (PMC5081052; doi:10.1093/hmg/ddw088)
Supplement: Supplementary Data [file supp_25_11_2360__index.html]

Very low-depth sequencing in a founder population identifies a cardioprotective APOC3 signal missed by genome-wide imputation — Very low-depth sequencing in a founder population identifies a cardioprotective APOC3 signal missed by genome-wide imputation — Supplementary Data 

# Very low-depth sequencing in a founder population identifies a cardioprotective *APOC3* signal missed by genome-wide imputation

## Supplementary Data

files

- Supplementary Data - pdf file
- Supplementary Data - pdf file
