# Supplementary material for: Very low-depth sequencing in a founder population identifies a cardioprotective APOC3 signal missed by genome-wide imputation
Source: Hum Mol Genet. 2016 May 4;25(11):2360–5. doi: 10.1093/hmg/ddw088 (PMC5081052; doi:10.1093/hmg/ddw088)
Supplement: Supplementary Data [file supp_ddw088_HMG-2015-D-00940-Suppl-Revision.pdf]

Figure S1. *APOC3* variants included in the burden test. Each line represents a labelled transcript (*APOC3-001* is annotated as canonical in Ensembl release 75, and is the only transcript included in the CCDS database(27)). Filled boxes correspond to coding sequence, hollow boxes to UTRs and lines to introns. Arrows denote the direction of transcription.

Table S1. Non-monomorphic variants from 1x sequencing data in the *APOC3* gene. Rsid is the variation identifier given by Ensembl VEP, chr: chromosome, position: position on chromosome, NEA: Non-effect Allele, EA: Effect Allele, MAF: minor allele in frequency estimated by GEMMA (in per cent, values of 0.04 indicate singletons), beta, s.e.: effect size and standard deviation for the effect allele calculated using GEMMA, p-value: score test p-value calculated using GEMMA.

rsid	chr	position	NEA	EA	MAF (%)	beta	s.e.	p-value
-	11	116700439	G	A	0.3	0.51	0.4	0.209
rs138617853	11	116700541	G	C	0.3	0.32	0.4	0.436
-	11	116700679	A	G	0.08	0.50	1	0.616
rs191196015	11	116700687	G	A	0.08	-0.17	1	0.867
rs12721090	11	116700775	C	T	0.8	0.12	0.22	0.588
rs618354	11	116700777	G	C	23.2	-0.03	0.04	0.555
rs11827682	11	116700785	C	T	0.8	0.12	0.22	0.588
rs734104	11	116700860	T	C	14.2	0.17	0.05	$4.08x10^{-3}$
-	11	116700919	G	T	0.08	-0.57	1	0.566
-	11	116700969	G	A	0.08	-0.32	1	0.752
rs2070669	11	116701122	G	C	34.3	0.07	0.04	0.080
rs2070668	11	116701153	T	G	49.3	-0.08	0.04	0.053
-	11	116701232	G	T	0.1	0.25	0.7	0.725
-	11	116701247	G	T	0.08	-0.45	1	0.650
-	11	116701259	T	C	0.08	-0.90	1	0.371
rs76353203	11	116701353	C	T	1.5	-1.09	0.16	$8.21x10^{-11}$
rs138326449	11	116701354	G	A	1.2	-1.18	0.18	$1.14x10^{-09}$
-	11	116701398	T	C	0.08	0.74	1	0.457
-	11	116701489	G	T	0.08	-0.07	1	0.942
rs4520	11	116701535	C	T	30.9	0.02	0.04	0.614
rs2070667	11	116701669	G	A	4.2	0.28	0.1	$6.09x10^{-3}$
rs2070666	11	116701674	T	A	19.6	-0.11	0.05	0.038
rs1269330	11	116701833	G	A	4.2	0.28	0.1	$6.09x10^{-3}$
-	11	116701848	C	A	0.2	0.44	0.44	0.321
rs5142	11	116701850	C	T	11.2	0.23	0.06	$4.89x10^{-4}$
rs12721099	11	116701898	T	C	4.2	0.28	0.1	$6.09x10^{-3}$
rs201477146	11	116702000	C	T	14.5	-0.11	0.06	0.073
-	11	116702014	T	C	0.08	0.73	1	0.469
rs187542976	11	116702066	T	C	0.3	-0.06	0.35	0.873
rs192473046	11	116702112	C	T	0.08	1.20	1	0.230
rs5141	11	116702123	C	T	10.9	0.23	0.06	$4.45x10^{-4}$
-	11	116702125	G	A	0.08	-1.77	1	0.0776

-	11	116702214	G	T	0.08	-0.18	1	0.855
rs12721098	11	116702229	C	T	1.7	-0.15	0.16	0.335
rs181006521	11	116702230	G	A	0.3	-0.13	0.4	0.754
-	11	116702239	C	A	0.08	0.92	1	0.355
-	11	116702242	A	G	0.08	-1.36	1	0.176
-	11	116702271	G	T	0.08	1.70	1	0.090
rs553080	11	116702345	T	C	4.2	0.27	0.1	$7.4x10^{-3}$
-	11	116702428	T	C	0.08	-1.34	1	0.180
rs5134	11	116702579	T	C	4.2	0.27	0.1	$7.4x10^{-3}$
-	11	116702586	C	A	0.1	-1.10	0.7	0.12
rs140223477	11	116702630	G	A	0.7	0.35	0.24	0.157
-	11	116702684	C	A	0.2	0.32	0.5	0.524
-	11	116702733	G	T	0.1	-0.76	0.7	0.285
-	11	116702734	G	T	0.08	0.00	1	0.999
-	11	116702761	G	T	0.08	-0.25	1	0.804
rs5132	11	116702778	C	T	3.4	0.32	0.11	$5.94x10^{-3}$
-	11	116702785	A	G	0.08	-0.26	1	0.792
-	11	116703037	G	T	0.1	-0.38	0.57	0.509
-	11	116703056	A	G	0.08	-0.18	1	0.853
-	11	116703099	C	A	0.1	-0.68	0.7	0.336
rs5130	11	116703146	T	C	15.1	0.27	0.05	$4.93x10^{-6}$
-	11	116703182	C	A	0.3	0.50	0.35	0.163
rs5128	11	116703640	C	G	10.9	0.23	0.06	$4.45x10^{-4}$
rs4225	11	116703671	G	T	49.8	-0.07	0.04	0.086
rs187628630	11	116703739	C	G	0.2	-0.37	0.44	0.408
