# Supplementary figures and images for: Very low-depth sequencing in a founder population identifies a cardioprotective APOC3 signal missed by genome-wide imputation
Source: Hum Mol Genet. 2016 May 4;25(11):2360–5. doi: 10.1093/hmg/ddw088 (PMC5081052; doi:10.1093/hmg/ddw088)

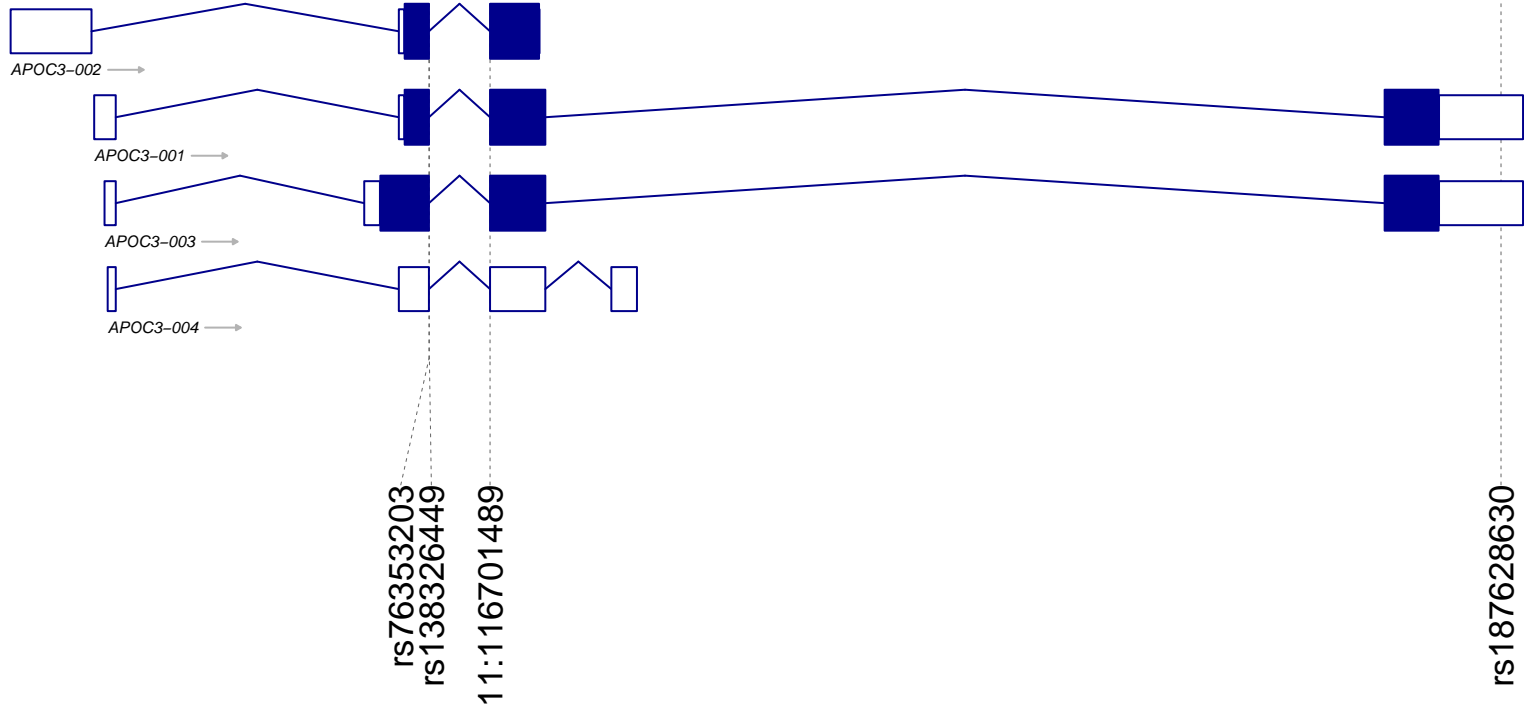

Supplement: Supplementary Data [file supp_ddw088_HMG_Gilly_FigureS1.pdf]
